# Genetic Diversity and Pedigree Analysis of Red Currant Germplasm

**DOI:** 10.3390/plants11131623

**Published:** 2022-06-21

**Authors:** Anna Pikunova, Svetlana Goryunova, Denis Goryunov, Olga Golyaeva, Maria Dolzhikova, Anna Pavlenko

**Affiliations:** 1 Russian Research Institute of Fruit Crop Breeding (VNIISPK), 302530 Orel, Russia; golyaeva@vniispk.ru (O.G.); dolzhikova@vniispk.ru (M.D.); tolpekina@vniispk.ru (A.P.); 2 Russian Academy of Sciences, Vavilov Institute of General Genetics, 119333 Moscow, Russia; svetlana.v.goryunova@gmail.com; 3 Russian Potato Research Center, 140051 Kraskovo, Russia; 4 Belozersky Institute of Physico-Chemical Biology, Lomonosov Moscow State University, 119992 Moscow, Russia; s.apocarpum@gmail.com

**Keywords:** *Ribes rubrum* L., red currant, varieties, breeding, germplasm, genetic collection, genetic diversity, genotyping-by sequencing, SNP markers, MDS analysis, admixture analysis

## Abstract

This represents the first report on the genetic diversity of red currant germplasm collections based on genotyping-by-sequencing (GBS) data. Genotypes of 75 individuals of different origin were assessed in more than 7.5K genome positions. Multidimensional scaling (MDS) analysis has been performed. There are five accessions that are significantly isolated from each other and from the rest of the analyzed cultivars. F_1_ offspring of *R. petraeum* Wulf (Rote Hollandische) and Gondouin, as well as Rote Spatlese (F_2_ of *R. petraeum* and F_2_ of *R. multiflorum* Kit.), are the most genetically isolated on the MDS plot. *Ribes multiflorum* is closer to the rest of cultivars than the three abovementioned accessions. Purpurnaya cultivar (F_1_ of Rote Spatlese) is located between Rote Hollandische and *R. multiflorum*. Other genotypes, mostly represented by varieties having several species in a pedigree, occupied the rest of MDS plot relatively evenly. Descendants of *R. multiflorum* have been placed in the left part of MDS plot, which underlines their genetic diversity from other accessions. White- and pink-fruited cultivars were clustered together, underlining genetic relatedness. Admixture analysis of GBS data reveals six clusters (K = 6). Presumably, clustering reflects relatedness to *R. petraeum*, *R. rubrum*, *R. vulgare* var *macrocarpum*, *R. multiflorum*, *R. vulgare,* and Jonker van Tets. Based on genotyping data, F_1_ offspring of *R. warscewiczs* Jancz (cultivar Viksne), *R. altissimum* Turcz (Cirald), and *R. palczewskii* (Jancz.) Pojark (Skorospelaya) have not exhibited strict separation and were placed in a pool with other varieties. This supports modern taxonomic classifications that do not consider *R. altissimum* and *R. palczewskii* as independent species.

## 1. Introduction

Red currant is a commercially important berry crop. Among other temperate climate crops, currant is relatively young. It has been cultivated for only 400–500 years. The first information about red currant appeared in Western Europe at the beginning of the 15th century in the explanatory dictionary of Dieffenbach, and in the 16th century it was widely used as a garden crop in Italy, France, and England [1]. The main red currant producers are Poland, Germany, Holland, Belgium, France, and Hungary. Germany and the Slovak Republic are leading producers of white currants [2].

Red currant belongs to the section Ribesia Berl of the *Ribes* genus (by Rehder, 1954) [3]. Ribesia Berl includes red currants originating from Europe (*R. rubrum* L.), garden currant (*R. vulgare* Lam.), many-flower currant (*R. multiflorum* kit.), rock red currant (*R. petraeum* Wulf.) (also found in northern Africa), and *R. pubescens* (Hedl.). In Eastern Siberia and the Far East, the Palchevsky currant (*R. palczewskii* (Jancz.) Pojark.), Varshevich currant (*R. warscewiczii* Jancz) (not found in wild form), dark purple currant (*R. atropurpureum* Mey), bristly currant (*R. hispidulum* (Jancz) Pojark.), Meyer currant (*R. meyeri* Maxim.), the highest currant (*R. altissimum* Turcz.), and a Chinese type of currant (*R. longeracemosum* Franch) are widespread [4].

Most modern cultivars of red currant are descendants of the following species: garden currant (*R. vulgare* Lam.), large-fruited variety (*R. vulgare* var. *macrocarpum*), red currant (*R. rubrum* L.), rock red currant (*R. petraeum* Wulf.), multi-flowered currant (*R. multiflorum* kit.), Varshevich currant (*R. warscewiczii* Jancz.), Palchevsky currant (*R. palczewskii* (Jancz.) Pojark and their hybrids. Other species and their hybrids are valuable as a source material for further breeding [4].

Genetic polymorphism and phylogeny of red currant with the use of DNA markers have been studied mainly in conjunction with other *Ribes* representatives such as black currant, gooseberry, etc. [5,6,7,8,9].

To date, according to published papers, currant germplasm collections have been genotyped using markers such as simple sequence repeats (SSRs) [8,9], Amplified Fragment Length Polymorphism (AFLP), chloroplast SSR (cp SSR) [6], inter-simple sequence repeats (ISSRs) [10], and random amplified polymorphic DNA (RAPD) [7,10] whereas none have been explored using up-to-date high-density genome-wide marker information, such as the SNP markers revealed by the genotyping-by-sequencing (GBS) approach.

For example, in the report of Palmieri et al. [9], 46 red currant cultivars in combination with black currant cultivars were characterized by 10 microsatellite markers. Based on the results, a dendrogram of genetic similarity was constructed. In the report of Mattia et al. (2008), AFLP and cpSSR markers were used to study 17 red currant cultivars along with wild populations of rock currant and gooseberry. A cluster analysis was performed [6]. The most wide-scale work was carried out by Antonius et al. [8], where 202 red currant cultivars from nine different European collections were analyzed by five microsatellites to create a non-centralized collection. However, the population structure of the red currant germplasm analysis has not been done in frame of the investigation conducted by Antonius et al.

Currently, microsatellite and SNP markers are preferred in the study of germplasm collections [11,12]. Larsen et al. (2018) [11] argue that GBS is superior to traditional SSR approaches because it allows detection of a much more detailed population structure and can be further exploited in genome-wide association studies (GWAS).

Thus, this is the first time the genetic diversity of red currant has been assessed by a high-throughput genotyping approach.

## 2. Results

### 2.1. Analysis of Pedigree Data

Seventy five genotypes were included in the analysis (Table 1).

**Figure 1:**
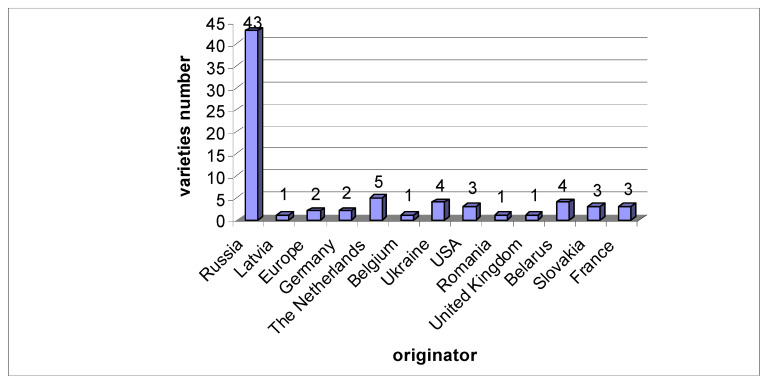
Countries of origin of the 73 cultivars involved in the analysis.

Most of the accessions (73) are the old and recent cultivars originating from different countries (Figure 1). The analysis included 43 cultivars of Russian breeding, originating from different breeding institutions in different climatic zones (Moscow, Saratov, Orel, Chelyabinsk, Novosibirsk, Southern Urals, etc.). In addition to the cultivars, one sample of the wild species *R. multiflorum* and one multi-species hybrid, 1426-21-80, were included in the analysis.

As far as was possible, pedigree data were collected from several generations up to the species (Table S1). In Table S2, based on the available pedigree data, we have listed which species are the ancestors of specific cultivars. Unfortunately, the pedigree of nine cultivars is completely unknown (Almaznaya, Belaya Krupnaya, Bulan Belaya, Novaya Krasnaya, Rachnovskaya, Rubin, Tatianina, Natali, Margaritar). For Losan (Chenonceau × Vierlandensky), the parents are known, but further their pedigrees are not known. For many cultivars, there are some ambiguities in the pedigrees, often on the paternal side. For example, Viksne is obtained from sowing seeds of *R. warscewiczs*, Transparent Blanche is described as a descendant of garden currant, and Rondom is obtained from pollination of *R. multiflorum* with a mixture of Versalskaya Krasnaya pollen and Rote Hollandische, etc.

According to pedigrees, both closely related cultivars and cultivars completely unrelated by pedigree were analyzed in this work. For example, cultivars Valentinovka, Dana, Dar Orla, Ogoniok, Orlovchanka, and Podarok Leta have been selected from the same hybrid family (Rote Spatlese × Jonkheer van Tets). In contrast, Rote Hollandische (*R. petraeum* × *R. rubrum*) and White Cherry (F1 of *R. vulgare*) have no common ancestors.

In pedigree analysis we used species names according to the classification of Rehder (1954) [3]. This classification is most widely used by red currant breeders. Analysis of pedigree revealed the following seven species to be ancestors of the analyzed genotypes: *R. rubrum* L., *R. vulgare* Lam., *R. petraeum* Wulf., *R. multiflorum* kit., *R. altissimum* Turcz., *R. warscewiczs* Jancz., and *R. palczewskii* (Jancz.) Pojark. According to the taxonomic classification of Rehder 1954 [3], all species except *R. altissimum* belong to Ribesia (Berl.) Zancz section of Ribesia Berl. subgenus of the *Ribes* L. genus. *R. altissimum* is absent in the classification of Rehder (1954).

Three species appeared only once in pedigree of the accessions; *R. altissimum*, according to the data of pedigrees, is the father of Cirald (Cirvya Pists (origin is not known) × *R. altissimum*); *R. warscewiczs* is present in the pedigree of Viksne, which is obtained from sowing seeds of Varshevich currant and differs in the cherry color of berries; Skorospelaya (Rannya Favorsky) was obtained in 1937 at the Suifuno-Ussuri fruit and berry experimental station (Primorsky Krai) from the seeds of *R. palczewskii*.

The remaining four species (*R. rubrum*, *R. petraeum*, *R. multiflorum*, *R. vulgare*) are widely represented by descendants in the first and more distant generations (up to the 6th).

**Figure 2:**
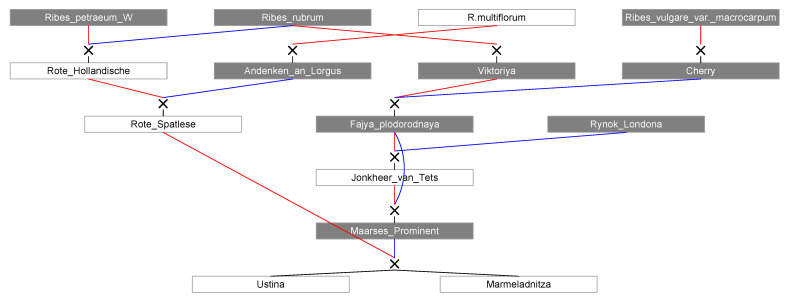
The pedigree of the cultivars Ustina and Marmeladnitza (in the white rectangle, the genotypes that are present in the analysis, in the gray-that are absent in the analysis, the red line is maternal, the blue line is paternal). The Figure is built using the Pedimap 1.2 software (© Plant Research International, 2004–2011 Roeland E. Voorrips, Wageningen, The Netherlands).

In addition to the descendants of *R. altissimum*, *R. warscewiczs*, and *R. palczewskii* mentioned above, only 13 cultivars have only one species in the pedigree; these are the 12 descendants of *R. vulgare* (including descendants of *R. vulgare* var. *macrocarpum*), and Kremovaya is a descendant of *R. rubrum*. Most cultivars (45) have several species in the pedigree: from two to four. For example, Marmeladnitza and Ustina have four species in their pedigree and represent the 3rd generation from rock red currant, the 3rd generation from red currant, the 3rd generation from multi-flowered currant, and the 4th generation from garden large-fruited currant (Figure 2).

The most numerous descendants are from *R. rubrum*. It has 43 cultivars: descendants in the 1st generation (Rote Hollandische, Kremovaya); in the 2nd generation, there are 12 cultivars (Tambovskaya Rannya, Rannya Sladkaya, Sakharnaya, Rote Spatlese, Belka, Vika, Mechta, Niva, Shedraya, Alfa, Gazel, Asya); in the 3rd generation, there are 18 cultivars (Jonkheer van Tets, Koral, Chelyabiskaya Sladkaya, Nadezhda, Pamyatnaya, Pamyat Gubenko, Uralskaya Krasnaya, Uralskie Zori, Uralsky Suvenir, Rovada, Marmeladnitza, Ustina, Bayana, Selyanochka, Blanka, Orlovskaya Zvezda, Osipovskaya, Purpurnaya), and in the 4th generation, there are 11 cultivars (Rolan, Svetlitza, Svyatomikhailovskaya, Alfa, Charodeyka, Valentinovka, Dana, Dar Orla, Ogonyok, Orlovchanka, Podarok Leta) (Figure S1).

Rock red currant (*R. petraeum*) is present in a pedigree of 31 cultivars, and it is also a significant part of the studied gene pool. There are cultivars from *R. petreum* in the 1st generation (Gondouin, Rote Hollandische), in the 2nd generation (12 cultivars: Tambovskaya Rannya, Rannya Sladkaya, Sakharnaya, Rote Spatlese, Belka, Vika, Mechta, Niva, Roza, Asya, Gazel, Koral), in the 3rd generation (18 genotypes: Marmeladnitza, Ustina, Alfa, Charodeyka, Bayana, Selyanochka, Blanka, Orlovskaya Zvezda, Osipovskaya, Purpurnaya, Rovada, Valentinovka, Dana, Dar Orla, Ogonyok, Orlovchanka, Podarok Leta, 1426-21-80), and in the 4th generation (Rolan) (Figure S2).

The analysis includes a representative of the wild species *R. multiflorum* as well as its descendants. *Ribes multiflorum* grows in southern and south-eastern Europe on the slopes of mountains and is distinguished by very long raceme on which there are up to 50 flowers [13]. F_1_ offspring of *R. multiflorum*-Rondom (obtained from the pollination of *R. multiflorum* with a mixture of Versal’skaya Krasnaya and Rote Hollandische pollen) and F_2_ offspring of *R. multiflorum*—Darnitza (Rondom × Altajskaya rannyaya) were analyzed. Andenken an Lorgus was obtained from *R. multiflorum* (not in the analysis), which became the father of Rote Spatlese (the 2nd generation from *R. multiflorum*, present in the analysis). Rote Spatlese inherited a long flower raceme. This variety is mainly of industrial use. It was widely used in breeding programs, including the VNIISPK breeding program [13]. Rote Spatlese is present in 18 analyzed genotypes, of which 17 are the 3rd generation from *R. multiflorum*, as well as the hybrid 1426–2180, which represents the 4th generation from *R. multiflorum* (Figure S3).

In addition to garden currant (*R. vulgare*), the pedigrees mention a garden large-fruited variety of currant (*R. vulgare* var. *macrocarpum*). In accordance with the pedigrees data, R.vulgare are presented in the pedigree of 15 cultivars: in the 1st generation, there are five cultivars (Gondouin, White cherry, Weisse Hollandische, North Star, Transparent Blanche), in the 2nd generation, there are six cultivars (Red Cross, Batishchevskaya, Shedraya, Niva, Orlovskaya Zvezda, Osipovskaya), and in the third generation, there are three cultivars (Krasnaya Andreichenko, Cascad, Belaya Potapenko) (Figure S4).

*R. vulgare* var. *macrocarpum* is present in the pedigree of 38 cultivars: the 1st generation is represented by two cultivars (Heros, Wagner’s Grape), the 2nd generation is represented by two cultivars (Red Cross, Nenaglyadnaya), the 3rd generation is represented by 13 cultivars (Jonkheer van Tets, Cascad, Krasnaya Andreychenko, Chelyabinsk Krasnaya, Shedraya, Rovada, Nadezhda, Pamytnaya, Pamyat Gubenko, Uralskaya Krasavitsa, Uralskiye Zori, Uralsky Suvenir, Belaya Potapenko), the 4th generation is represented by 11 cultivars (Rolan, Alpha, Charodeyka, Valentinovka, Dana, Dar Orla, Ogonyok, Orlovchanka, Podarok Leta, Svetlitza, Svyatomikhaylovskaya), the 5th generation is represented by four cultivars (Marmeladnitza, Ustina, Asya, Gazel), and the 6th generation is represented by hybrid 1426-21-80 (Figure S5).

### 2.2. Study of the Genetic Diversity of Red Currant

The genotypes of 75 individuals were assessed by genotyping-by-sequencing (GBS) data. In total, more than 8.5K biallelic SNPs were discovered. Among the variants there were 5249 transitions and 3334 transversions, i.e., transition to transversion ratio was 1.57. Mean read depth (DP) composed around 7.92. Positions with a high proportion (>89.5%) of missing calls were filtered out. Subsequently, the VCF file with genotyping data contained 7674 variants.

#### 2.2.1. Multidimensional Scaling (MDS) Analysis

**Figure 3:**
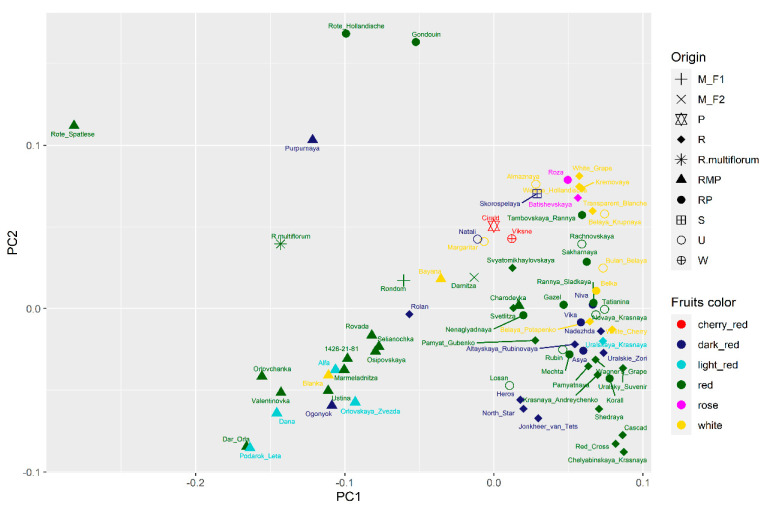
MDS plot based on GBS data on polymorphism of 76 genotypes from the VNIISPK collection of red currant. M_F1—F_1_ descendant of *R. multiflorum*; M_F2—F_2_ descendant of *R. multiflorum*; P—F_1_ descendant of *R. altissimum*; R—descendant of *R. rubrum*; RMP—descendant of *R. rubrum*, *R. multiflorum* and. *R. petraeum*; RP—descendant of *R. rubrum* and *R. petraeum*; S—descendant of *R. palczewskii*; U—origin is unknown, W—descendant of *R. warscewiczs*.

Multidimensional scaling (MDS) analysis was performed based on this GBS data (Figure 3).

F_1_ offspring of *R. petraeum*-Rote Hollandische and Gondouin as well as F_2_ *R. petraeum* descendent-Rote Spatlese (Rote Hollandische × Andenken an Lorgus) are the most genetically isolated on the MDS plot. *R. multiflorum* is closer to the major samples group than three abovementioned accessions. Purpurnaya cultivar (F_1_ of Rote Spatlese) is located between Rote Hollandische and *R. multiflorum*.

The remaining 70 genotypes are grouped together, relatively evenly distributed over an area resembling a triangle.

In the lower right corner of this triangle there is a group of two cultivars bred in the USA (Cascad, Red Cross) and the Russian cultivar Chelyabinskaya Krasnaya, of unknown origin. The group in the upper corner is formed mainly by white and pink cultivars. The lower left corner is occupied by closely related cultivars Dar Orla and Podarok Leta of VNIISPK breeding (Russia), originating from the crossing of Rote Spatlese × Jonkheer van Tets.

#### 2.2.2. Admixture Analysis

**Figure 4:**
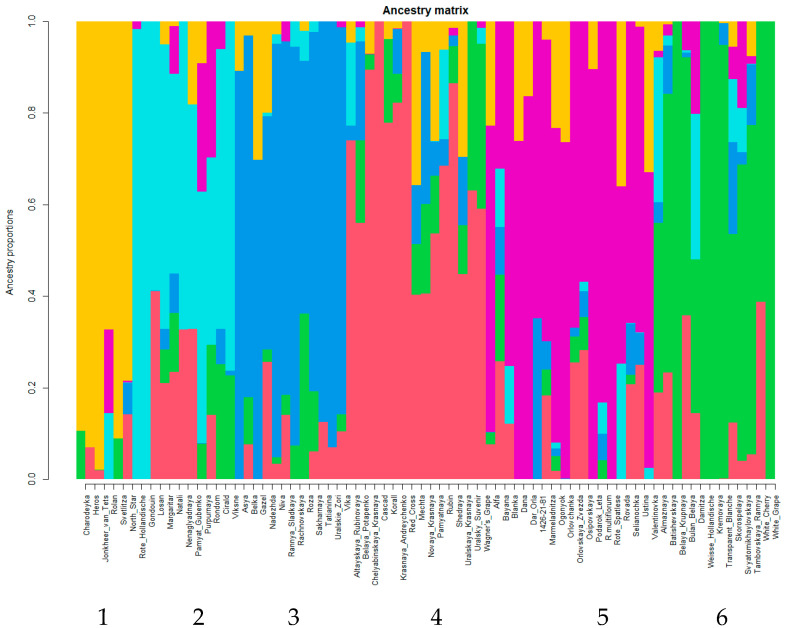
Population structure for 75 red currant genotypes. Colors represent different assigned clusters. The *x*-axis provides accession names and respective assigned cluster whereas the *y*-axis provides the probability of each accession belonging to the assigned cluster. Numbers in the bottom of figure correspond to cluster’s numbers presented in the text.

Determination of the appropriate number of clusters (K) based on the cross-entropy criterion by LEA software resulted in K = 6 (Figure S6, Figure 4).

The first cluster includes six cultivars, four of which are related by pedigree—Jonkheer van Tets and its three F_1_ offspring (Charodeyka, Rolan, Svetlitza). Jonkheer van Tets pedigree is distinct from other analyzed genotypes by paternal line Rynok Londona, of which the pedigree is unknown. Interestingly, according to pedigree data, 11 analyzed cultivars are F_1_ offspring of Jonkheer van Tets, but only three of them related to Jonkheer van Tets by maternal line are clustered together with it. Another two genotypes in this cluster are Heros (bred at Germany, F_1_ descendant of *R. vulgare*. var *macrocarpum*) and North Star (bred at USA, F_1_ descendant of *R. vulgare*).

The second cluster includes 11 cultivars, and for three of them the pedigrees are not known. Five cultivars are F_1_ (Gondouin, Rote_Hollandische), F_2_ (Nenaglyadnaya, Rondom), F_3_ (Purpurnaya) descendants of R. petraeum. No obvious relatedness by pedigree was revealed for Viksne (F_1_ of *R. warchevichii*), Cirald (F_1_ of *R. altissimum*) and Pamyat_Gubenko (Fajya plodorodnaya ×?). Interestingly, Nenaglyadnaya and Rondom obtained from pollination by pollen mixture and one of possible pollen donors is Rote Hollandische. Admixture analysis placed Nenaglyadnaya and Rondom together with Rote Hollandische. Thus, we presume that this underlies their relatedness and clarifies pedigree.

In the third cluster, 10 out of 12 cultivars are F1 and F2 descendants of *R. rubrum*. For two cultivars the pedigrees are not known.

Cluster 4 comprised a set of 15 cultivars. For two cultivars, pedigrees are not known. Eleven cultivars are obviously related through the pedigree as F_1_, F_2_ and F_3_ descendants of *R. vulgare* var. *macrocarpum*. There are no obvious pedigree relations of Mechta (CHulkovskaya × *) and Korall (Pervenec × Tambovskaya_Rannya) to other genotypes of this cluster. It is worthwhile to mention that Mechta is very admixed and less than half of its column is colored in cluster color.

The fifth cluster includes 18 cultivars. They are *R. multiflorum* and its progeny. Precisely speaking, not all descendants of *R. multiflorum* are in this cluster. Four (Darnitza, Rondom, Rolan, Purpurnay) out of 21 *R. multiflorum* descendants are placed in different clusters, possibly due to another ancestors.

The sixth cluster includes 13 cultivars. Eight of them are white-fruited and one has pink-colored berries. Four cultivars with a white color of berries are F_1_ offspring of *R. vulgare*. Other four white-fruited cultivars have no pedigree data. This cluster included eight out of 13 white-fruited cultivars analyzed in this study. Perhaps they are most close to *R. vulgare*. Besides white-and pink-fruited cultivars, the sixth cluster includes four red-fruited cultivars. Two of them, Darnitza and Svyatomikhaylovskaya, are related to each other by father (Altajskaya rannyaya, not analysed, pedigree unknown). Other red-fruited cultivars are Tambovskaya Rannya and Skorospelaya. There is no obvious pedigree relatedness between them and other samples of this cluster.

## 3. Discussion

This work is devoted to the study of the genetic diversity of the red currant germplasm. In the classical breeding approach, genetic diversity is considered on the basis of phenotype and pedigree data. Pedigree data is often unknown, incomplete, or may contain errors. In this regard, relying solely on the data of pedigrees, it is difficult to analyze the genetic diversity of the red currant germplasm.

Unstable taxonomic classification within the currant genus also contributes its share of ambiguity. For example, different numbers of species within the genus are distinguished in different years, and recently there has been a tendency to enlarge species [14] as sometimes a species is recognized as a synonym of another species or renamed into a variety of other species, or vice versa. For example, Palczewski currant was previously considered as a variety of red currant *Ribes rubrum* var. *palczewskii* JANCZ [15]. Currently, it is also noted that a number of species, including Palchevsky currant, have an unclear systematic position [16].

For the seven species involved in the origin of the genotypes included in this work, we analyzed two online databases of modern taxonomy—Plants of the World Online (POWO) [17] and World Flora online (WFO) [18]. They are united in relation to the species *Ribes rubrum* L., *Ribes petraeum* Wulf, and *R. multiflorum* Kit. that are accepted as species. Both databases refer to *Ribes vulgare* Lam. as a synonym of *Ribes rubrum* L. and *Ribes palczewskii* (Jancz.) Pojark. as a synonym of *Ribes spicatum* subsp. *lapponicum* Hyl. and *R. altissimum* Turzs. as a synonym of *Ribes petraeum*. The taxonomic position of *R. warszewiczii* Jancz. is uncertain. According to the web resource of the International Dendrology Society, *Ribes warszewiczii* was described in 1904 from a plant growing in the Botanic Garden at Krakow, Poland, said to have been raised forty years previously from seeds received from Siberia. It has larger flowers than in *R. spicatum*, is pinkish with a suggestion of a disk in the receptacle, is borne in pendulous racemes, and has large, acid fruits darker in color than those of a Morello cherry [19]. World Flora online states that *Ribes warszewiczii* Jancz. ex Vilm. and Bois was unchecked by WFO and awaiting taxonomic scrutiny. A POWO database search for *Ribes warszewiczii* has shown no results. Thus, from the point of view of modern taxonomic classifications, four species participate in the pedigrees of analyzed cultivars (in addition to *Ribes warszewiczii*, for which status is uncertain): *Ribes rubrum* L., *Ribes petraeum* Wulf, *R. multiflorum* Kit., *Ribes spicatum* subsp. *lapponicum* Hyl. Thus, in this work, among all the analysed accessions, there are 26 *Ribes rubrum* offspring, 14 *Ribes rubrum* × *Ribes petraeum* hybrids, 19 accessions containing all the three species i.e., *R. petraeum*, *R. multiflorum, R. rubrum* simultaneously in the pedigree, one *R. spicatum* descendent and one descendent of *Ribes warszewiczii* and two *R. multiflorum* F_1_ (Rondom) and F_2_ offspring (Darnitza). Rondom and Darnitza possible have also got *R. rubrum* and *R. petraeum* in a pedigree. The pedigree of 10 accessions is currently unknown.

Old cultivars Gondouin (*R. petraeum* × *R. vulgare*) and Rote Hollandische (*R. petraeum* × *R. rubrum*), which are F_1_ descendants of *R. petraeum*, occupy the topmost position on the graph, and they are significantly genetically differ from other genotypes. It is likely that it was the proximity to rock red currant that caused the significant difference between Gondouin and Rote Hollandische from other cultivars.

In the work of Mattia (2008) [6], the analysis of the polymorphism of AFLP and cpSSR markers was used to study 17 cultivars of red currant (rock currant was also present in the pedigree of some of them) and two wild populations of rock currant and gooseberry. Clustering of the dendrogram with high bootstrap support separates cultivars from wild populations of rock currant. So, in our work, cultivars closer to the wild species descendants of the first generation of *R. petraeum* are most different from other cultivars, and this species is already “significantly diluted”.

At the same time, Cirald, having *Ribes petraeum (R. altissimum)* as a paternal parent, was not genetically separated from the main sample group on the plot. Additional data are probably required to confirm whether *Ribes petraeum* is a parent of the Cirald.

The descendants in the 1st generation from *R. warszewiczii* (Viksne) and *R. palczewskii* (Skorospelaya) are placed quite close to each other, and on the other hand do not show significant differences from modern cultivars; they are placed among the descendants of *Ribes rubrum* and interspecific hybrids. This may illustrate differences in speciation and also reflect subjectivity of taxonomy. Separation of species was historically based on morphological differences that do not always have an equal degree of difference between genomes.

Almost all descendants of *R. multiflorum* are located in the left part of the MDS plot. Only Charodeyka (Jonkheer van Tets × Rote Spatlese) turned out to be closer to other cultivars obtained from *R. rubrum* and *R. rubrum* and *R. petraeum* interspecific hybrids, without the participation of *R. multiflorum*. Charodeyka, according to the pedigree, is related to *R. multiflorum* in paternal form.

We would like to note that according to the literature, when checking pedigrees, including fruit and berry crops, with the use of microsatellite loci analysis, errors in pedigrees are not infrequently detected [20,21,22,23,24]. For example, when clarifying the origin of apple cultivars bred in Dresden-Pillnitz, according to microsatellite loci, it was revealed that ‘Pimona’ and ‘Pikora’ originated from a different cultivar than the intended pollen donor [20]. In the work of Evans et al. (2011), in a study of the apple germplasm using microsatellite analysis for 12 accessions, only one of the two reported parents could be confirmed, and their other parent was inconsistent with the marker data [21]. In the work of Pikunova et al., the parentage of black currant cultivar Ocharovanie (1168 × Ekzotika) is questioned as = Ekzotika and Ocharovanie had no common alleles in three SSR loci (e4-D03, g1-E03, g2-B20). It is probably due to pollination by other pollen [23]. In the work of Girichev et al. (2017), 39 *Rubus* cultivars were tested on trueness-to-type using pedigree information and the SSR fingerprints. Six cultivars were found for which the female parent could not be confirmed and for eight cultivars the male parent could not be confirmed [24]. Nine parentages were completed or revised during study of grapes by means of SSR markers. For example, Manzoni crosses 2–14 and 2–15 that were reported by the breeder as ‘Cabernet Sauvignon × Prosecco’ actually have ‘Cabernet franc’ instead of ‘Cabernet Sauvignon’ as a parent. ‘Covè’, reported as a cross of ‘Harslevelu £ Malvasia bianca lunga’ resulted instead from a selfing of ‘Harslevelu’ [22].

In our work, Charodeyka was separated from the rest of *R. multiflorum* descendants in the MDS plot. It was probably not obtained from the pollen of Rote Spatlese, and thus is not related to *R. multiflorum*. However, this hypothesis needs further research. For a clear comparison and reconstruction of the flow of alleles from parents, it is desirable to analyze both parental forms and the Charodeyka by polymorphism of microsatellite loci.

Admixture analysis of GBS data reveals six clusters. Admixture is present in all clusters that underline the complicated pedigree of most of analyzed cultivars. Inside of clusters most of the samples are related by pedigree. Presumably, clustering reflects relatedness to *R. petraeum* (Cluster 2), *R. rubrum* (Cluster 3), *R. vulgare* var *macrocarpum* (4), *R. multiflorum* (5), *R. vulgare* (6) and Jonker van tets (1). F_1_ of *R.altissimum* Turcz and F_1_ of *R. warscewiczs* Jancz. are situated inside of *R. petraeum* related cluster. F_1_ of *R. palczewskii* (Jancz.) is situated inside of *R. vulgare* related cluster.

MDS and admixture analyses of GBS data complement each other. On MDS, plot cultivars from one admixture cluster are situated mostly together but form spots of different areas (Figure S7). For example, cultivars of the 5th cluster (*R. multiflorum* and its descendants) occupy nearly half of the MDS plot. Cultivars of the 3rd, 4th, and 6th clusters formed relatively compact spots on MDS plot.

**Figure 5:**
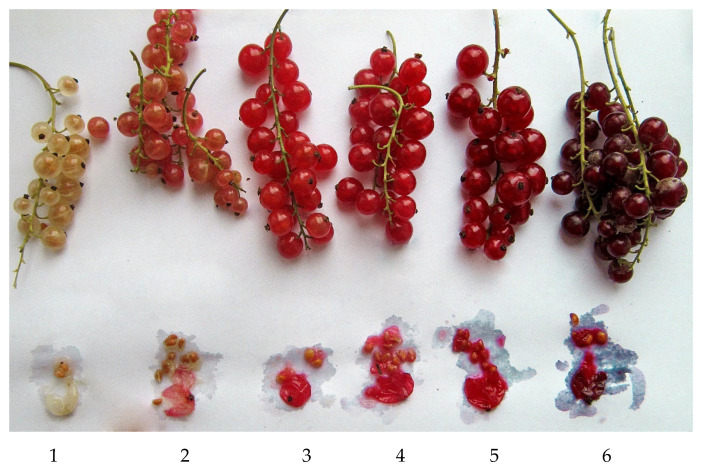
Variety of berry color of red currant cultivars (1—Weisse Hollandische, 2—Batishevskaya, 3—hybrid form (not included in the analysis), 4—Pamyat Gubenko, 5—Jonkheer van Tets, 6—Viksne).

Grouping associated with berry color is observed based on genotyping data. The berry color of red currant cultivars varies from white to cherry (Figure 5).

Two cultivars with cherry-colored fruits were included in the study (Cirald and Viksne). These accessions were located next to each other on the scatter plot. According to the pedigree data, they were not related and derived from two different species. At the same time, the revealed similarity in the fruit color and the high homogeneity on a genetic level of Cirald and Viksne may suggest their joint origin. Therefore, additional studies of these samples are required.

As is known, white-fruited cultivars exist in red currant germplasm. This trait is recessive and is sometimes considered as a consequence of a single mutation [25]. There are also pink cultivars that differ from white ones by the presence of some color in the peel of the fruit, but not in the pulp (see Figure 4), while the red cultivars have intensely colored berry peel and pulp. Our work presents 13 cultivars with white coloring (Blanka, Transparent Blanche, White Cherry, Bayana, Belaya Potapenko, Belka, Weisse Hollandische, Kremovaya, White Grape, Almaznaya, Belaya Krupnaya, Boulogne Blanche, Margaritar) and two cultivars with pink coloring (Batishevskaya, Roza). It is interesting that all of them except for Blanka are in the MDS plot in the upper part of the main cultivars group (Figure 4). The separation of Blanka is difficult to explain. The analysis of pedigrees of white- and pink-fruited cultivars (15 in total) showed that the origin of four cultivars is not known (Almaznaya, Belaya Krupnaya, Boulogne Blanche, Margaritar), six cultivars are descendants of garden currant (in the 1st generation—Transparent Blanche, White Cherry, White Grape, Weisse Hollandische; in the 2nd—Batishevskaya, (Myasokrasnaya × White Grape); and in the 3rd—Belaya Potapenko (Red Cross (Cherry × White Grape) × Krasnaya Sibiryachka (Red Cross × Red Cross)). The remaining five cultivars in the pedigrees have no direct indication of a connection with garden currant, but they all have unknown origin of the paternal form, namely, Blanka, Bayana, and Belka have a common father—Red Lake, a cultivar of unknown origin, and the origin of the father of Roza (Chulkovskaya (*R. rubrum* × *R. petraeum*) × Rose Cheer (unknown)) is also not known, but Kremovaya was obtained from open pollination of a red currant form. According to population structure analysis, eight white-fruited cultivars are situated in one cluster together with one pink-and four red-fruited cultivars. Four white-berried cultivars are F_1_ descendants of *R. vulgare* (Transparent Blanche, White Cherry, White Grape, Weisse Hollandische). For the other four white-fruited cultivars of this cluster, pedigree is unknown. Perhaps these eight cultivars are closest to *R. vulgare* than the other five white-fruited cultivars, possibly representing more distinct generations from *R. vulgare.*

The analyzed cultivars with light color (white and pink) apparently have a common origin, which is consistent with our GBS-data and SSR data [9], where all the white currant accessions plus the single pink colored variety clustered together (subcluster 5, 12 cultivars) in a dendrogram constructed on the basis of polymorphism of microsatellite loci [9]. Similarly, in the report of Lanham and Brennan 1998 [26] «Characterization of the genetic resources of redcurrant (*Ribes rubrum*: subg. Ribesia) using anchored microsаtellite markers», the authors note that although white currants are a color variant of red currants, rather than a separate taxonomic group, all three white currant cultivars are grouped tightly together. In this study, based on the known data of pedigrees of six of the 15 genotypes, it can be assumed that the common origin is inherited from *R. vulgare*.

In the opposite way, among raspberries, a number of yellow fruited cultivars were dispersed on three different clusters, suggesting a convergent evolution of this trait [24].

From the point of view of the mutant nature of the genetic difference of genomes between red and white cultivars, genetic difference should be insignificant, but on the other hand, from the point of view of the common origin of white–fruited cultivars from common ancestor, the connection can be seen, which is observed in our studies and other reports [9,26].

Cultivars with unknown origin (Losan, Novaya Krasnaya, Rubin, Tatianina, Natali, Almaznaya, Belaya Krupnaya, Bulan Belaya, Margaritar) are placed in the right part of the lower triangle of the MDS plot. According to the logic of genotype distribution, it can be assumed that they are not descendants of the multi-flowered currant. For white-fruited cultivars (Almaznaya, Belaya Krupnaya, Bulan Belaya, Margaritar), the presence of *R. vulgare* in the genome is likely. According to admixture clustering, Novaya Krasnaya and Rubin possibly have *R. vulgare* var *macrocarpum* in a pedigree. Losan, Natali, and Margaritar possibly have *R. petraeum* in a pedigree. Rachnovskaya and Tatianina possibly have *R. rubrum* in a pedigree.

Thus, it has been shown that majority of the red currant cultivars have a high degree of genetic similarity, but several genetically distinct cultivars were revealed simultaneously. The latter were the result of interspecific hybridization. Hence, there is a potential for a significant extension of the genetic diversity of red currant and the application of interspecific crosses to further crop improvement.

## 4. Materials and Methods

Seventy-five samples used for this study are listed in Table 1. They were obtained from the Russian Research Institute for Fruit crop breeding (Oryol, Russia) germplasm collection. DNA isolation was performed on young leaves according to Doyle and Doyle (1990) [27]. The quality of the purified DNA samples and DNA concentration were assessed by gel electrophoresis and Qubit 3.0 Fluorometer (ThermoFisher Sscientific, Waltham, MA, USA). GBS was performed according to Poland et al., 2012 [28]. GBS-library was sequenced on Illumina NovaSeq 6000 in SE100 mode.

As a result of sequencing, an average of 1.5 million readings per sample were obtained.

Sequencing reads were used for variants calling in the Tassel UNEAK pipeline [29]. General genotyping statistics over the VCF file were assessed via vcfstats tool (https://github.com/pwwang/vcfstats accessed on 19 May 2022). In total, more than 8.5K biallelic SNPs were discovered. Positions with a high proportion (>89.5%) of missing calls were filtered out. MDS analysis of the genotypes was accomplished in Tassel 5 [30] software (version 5.2.80) and visualized in ggplot2 [31] (version 3.3.5) and ggrepel (https://github.com/slowkow/ggrepel accessed on 5 April 2022) *R libraries.* Admixture analysis was performed in the LEA R package. [32]. The number of K populations was assessed from 1 to 12 clusters, with 100 replications accomplished for each K value. The best K value was selected based on the cross-entropy criterion.

For pedigree visualization, Pedimap 1.2 software [33] has been used. The data file for Pedimap is represented in supplementary materials Table S1.

## 5. Conclusions

This work is devoted to the study of the genetic diversity of red currants by a high-throughput genotyping approach in combination with the analysis of pedigrees. It was revealed that pedigree data of red currant accessions are often unknown, incomplete, or contain errors. Analysis of pedigrees revealed seven species to be ancestors of analyzed genotypes (*R. rubrum* L., *R. vulgare* Lam., *R. petraeum* Wulf., *R. multiflorum* kit., *R. altissimum* Turcz., *R. warscewiczs* Jancz., *R. palczewskii* (Jancz.) Pojark). From the point of view of modern taxonomic classifications, four species participate in the pedigrees of analyzed cultivars (in addition to *R. warszewiczii*, for which the status is uncertain): *R. rubrum* L., *R. petraeum* Wulf, *R. multiflorum* Kit., *R. spicatum* subsp. *lapponicum* Hyl.

This represents the first study of red currant genetic diversity based on high-throughput genotyping data. Multidimensional scaling (MDS) analysis of the genotyping data was performed based on more than 7.5 K snp markers. F_1_ offspring of *R. petraeum* Wulf. (Rote Hollandische and Gondouin); Ribes multiflorum; Rote Spatlese (F_2_ of *R. petraeum* and F_2_ of *R. multiflorum* Kit.); and Purpurnaya (F_1_ of Rote Spatlese) are significantly isolated from each other and the rest of the analyzed cultivars. It is likely that it was the proximity to rock red currant that caused the significant difference between Gondouin and Rote Hollandische and other cultivars. Descendants of *R. multiflorum* form a separate group close to *R. multiflorum* on the MDS plot, which underlines their genetic relatedness.

Admixture analysis of GBS data reveals six clusters. Inside of the clusters, most samples were related by pedigree. Presumably, clustering reflects relatedness to *R. petraeum*, *R. rubrum*, *R. vulgare* var *macrocarpum*, *R. multiflorum*, *R. vulgare,* and Jonker van Tets.

Based on GBS data, F_1_ offspring of *R. warscewiczs* Jancz. (cultivar Viksne), *R. altissimum* Turcz. ex Pojark. (Cirald), and *R. palczewskii* (Jancz.) Pojark. (cultivar Skorospelaya) have not exhibited strict separation and were placed in a pool with other varieties. This supports modern taxonomic classifications that do not consider *R. altissimum* and *R. palczewskii* as independent species.

White- and pink-fruited cultivars were clustered together, underlining genetic relatedness.

GBS analysis is a powerful approach for assessing the genetic diversity of germplasm collections based on revealed SNP markers. Data obtained could be used for the evaluation of assortment and breeding strategy and further improvement of the crop.

## Figures and Tables

**Table 1:** Plant material.

No.	Cultivar	Originator	Parentage
1	1426-21-80	Russia, VNIISPK, Orel	82-4-11 (Rote Spatlese × Chulkovskaya) × 78-2-118 (Rote Spatlese × Maarses Prominent)
2	Alfa	Slovakia	Jonkheer van Tets × Rote Spatlese
3	Almaznaya (Belaya Fajya)	VSTISP, Moscow, Russia	unknown
4	Altayskaya Rubinovaya	Lisavenko NIISS, Barnaul, Russia	open pollination of Fajya plodorodnaya
5	Asya	VNIISPK, Orel, Russia	Chulkovskaya × Maarses Prominent
6	Batishevskaya	Belarus	Myasokrasnaya × White Grape
7	Bayana	VNIISPK, Orel, Russia	Rote Spatlese × Red Lake
8	Belaya Krupnaya	Belarus	unknown
9	Belaya Potapenko	Novosibirsk, Russia	Red Cross × Krasnaya Sibiryachka
10	Belka	VNIISPK, Orel, Russia	Chulkovskaya × Red Lake
11	Blanka	Slovakia	Rote Spatlese × Red Lake
12	Bulan Belaya (Boulogne Blanche)	France	unknown
13	Cascad	USA	open pollination of Diploma
14	Charodeyka	Lviv, Ukraine	Jonkheer van Tets × Rote Spatlese
15	Chelyabinskaya Krasnaya	Russia	open pollination of Fajya Plodorodnaya
16	Cirald	N. I. Vavilov All-Russian Institute of Plant Genetic Resources (VIR), St. Petersburg, Russia	Cirv’ya Pists × *R. altissimum*
17	Dana	VNIISPK, Orel, Russia	Rote Spatlese × Jonkheer van Tets
18	Dar Orla	VNIISPK Orel, Russia	Rote Spatlese × Jonkheer van Tets
19	Darnitza	Ukraine	Rondom × Altayskaya Rannya
20	Gazel	VNIISPK, Orel Russia	Chulkovskaya × Maarses Prominent
21	Gondouin	Belgium	*Ribes petraeum* × *Ribes vulgare*
22	Heros	Germany	F1 of *Ribes vulgare* var. *macrocarpum*
23	Jonkheer van Tets	The Netherlands	Fajya plodorodnaya × Rynok Londona
24	Korall	Saratov, Russia	Pervenec × Tambovskaya Rannya
25	Krasnaya Andreychenko	Novosibirsk, Russia	open pollination of Red Cross
26	Kremovaya	Research Institute of Genetics and Breeding of Fruit Plants, Michurinsk, Russia	open pollination of *Ribes rubrum*
27	Losan	Slovakia	Chenonceau × Vierlandensky
28	Margaritar	Romania	unknown
29	Marmeladnitza	VNIISPK, Orel, Russia	Rote Spatlese × Maarses Prominent
30	Mechta	South Ural Research Institute, Chelyabinsk, Russia	open pollination of Chulkovskaya
31	Nadezhda	Russia	open pollination of Fajya Plodorodnaya
32	Natali	Russia	unknown
33	Nenaglyadnaya	Belarus	Cherry pollinated by mix of pollen of Chudesnica and Rote Hollandische
34	Niva	VNIISPK, Orel, Russia	Minnesota × Chulkovskaya
35	North Star	USA	F1 of *Ribes vulgare*
36	Novaya Krasnaya	Russia	unknown
37	Ogonyok	VNIISPK, Orel, Russia	Rote Spatlese × Jonkheer van Tets
38	Orlovchanka	VNIISPK, Orel, Russia	Rote Spatlese × Jonkheer van Tets
39	Orlovskaya Zvezda	VNIISPK, Orel, Russia	Rote Spatlese × Minnesota
40	Osipovskaya	VNIISPK, Orel, Russia	Rote Spatlese × Minnesota
41	Pamyat Gubenko	South Ural Research Institute, Chelyabinsk, Russia	open pollination of Fajya plodorodnaya
42	Pamyatnaya	South Ural Research Institute, Chelyabinsk, Russia	open pollination of Fajya Plodorodnaya
43	Podarok Leta	VNIISPK, Orel, Russia	Rote Spatlese × Jonkheer van Tets
44	Purpurnaya	Belarus	openpollination of Rote Spatlese
45	*R. multiflorum*	-	*R.multiflorum*
46	Rachnovskaya	VSTISP, Moscow, Russia	unknown
47	Rannya Sladkaya	VSTISP, Moscow, Russia	Chulkovskaya × Laturnajs
48	Red Cross	USA	Cherry × White Grape
49	Rolan	The Netherlands	Jonkheer van Tets × Rozetta
50	Rondom	The Netherlands	*R. multiflorum* pollinated by mix of pollen Versal’skaya Krasnaya and Rote Hollandische
51	Rote Hollandische	France	*Ribes rubrum* × *Ribes petraeum*
52	Rote Spatlese	Germany	Rote Hollandische × Andenken an Lorgus
53	Rovada	The Netherlands	Fajya Plodorodnaya × Rote Spatlese
54	Roza	Russia, VNIISPK, Orel	CHulkovskaya × Rose Cheer
55	Rubin	Russia	unknown
56	Sakharnaya	Russia	Chulkovskaya × Laturnajs
57	Selianochka	VNIISPK, Orel, Russia	Rote Spatlese × Red Lake
58	Shedraya	Russia	Fajya Plodorodnaya × Zamok Hauton
59	Skorospelaya (Rannya Favorskoy)	Russia	F 1 of *R. palczewskii*
60	Svetlitza	Lviv, Ukraine	Jonkheer van Tets × Fertodi Piros
61	Svyatomikhaylovskaya	Institute of Horticulture UААS, Ukraine	Jonkheer van Tets × Altayskaya Rannya
62	Tambovskaya Rannya	Russia	Mestnaya Krasnaya × Rote Hollandische
63	Tatianina	Russia	unknown
64	Transparent Blanche	France	F1 of *Ribes vulgare*
65	Uralskaya Krasnaya	South Ural Research Institute, Chelyabinsk, Russia	open pollination of Fajya Plodorodnaya
66	Uralskie Zori	South Ural Research Institute, Chelyabinsk, Russia	open pollination of Fajya Plodorodnaya
67	Uralsky Suvenir	South Ural Research Institute, Chelyabinsk, Russia	open pollination of Fajya Plodorodnaya
68	Ustina	VNIISPK, Orel, Russia	Rote Spatlese × Maarses Prominent
69	Valentinovka	VNIISPK, Orel, Russia	Rote Spatlese × Jonkheer van Tets
70	Vika	VNIISPK, Orel, Russia	Chulkovskaya × Red Lake
71	Viksne	Latvia	F1 of *R. warscewiczs*
72	Wagner’s_Grape	Europe	F1 of *Ribes vulgare* var. *macrocarpum*
73	Weisse Hollandische	The Netherlands	F1 of *Ribes vulgare*
74	White Cherry	Europe	F1 of *Ribes vulgare*
75	White Grape	United Kingdom	F1 of *Ribes vulgare*

## Data Availability

Reads form GBS data are downloaded at NCBI database, PRJNA850177, https://www.ncbi.nlm.nih.gov/sra/PRJNA850177 accessed on 5 May 2022.

## Supplementary Materials

The following supporting information can be downloaded at: https://www.mdpi.com/article/10.3390/plants11131623/s1, Figure S1: *Ribes rubrum* descendants. White rectangles show presented in this study genotypes. Grey rectangles show absented in this study genotypes. Figure has been created by Pedimap software; Figure S2: *Ribes petraeum* descendants. White rectangles show presented in this study genotypes. Grey rectangles show absented in this study genotypes. Figure has been created by Pedimap software; Figure S3: *Ribes multiflorum* descendants. White rectangles show presented in this study genotypes. Grey rectangles show absented in this study genotypes. Figure has been created by Pedimap software; Figure S4: *Ribes vulgare* descendants. White rectangles show presented in this study genotypes. Grey rectangles show absented in this study genotypes. Figure has been created by Pedimap software; Figure S5: *Ribes vulgare* var *macrocarpum* descendants. White rectangles show presented in this study genotypes. Grey rectangles show absented in this study genotypes. Figure has been created by Pedimap software; Figure S6: Determination of the appropriate number of clusters (K) based on the cross-entropy criterion by LEA software; Figure S7: Admixture clusters designated on MDS plot. Numbers refer to population structure clusters. Table S1: Pedigree data of analyzed genotypes and their ancestors until wild species (may be used for Pedimap software application); Table S2: Ancestors species in analyzed genotypes pedigrees.
