# Supplementary material for: Genetic Diversity and Pedigree Analysis of Red Currant Germplasm
Source: Plants (Basel). 2022 Jun 21;11(13):1623. doi: 10.3390/plants11131623 (PMC9269202; doi:10.3390/plants11131623)
Supplement: Supplementary file 1 [file plants-11-01623-s001.zip › Figures S1-7.pdf]

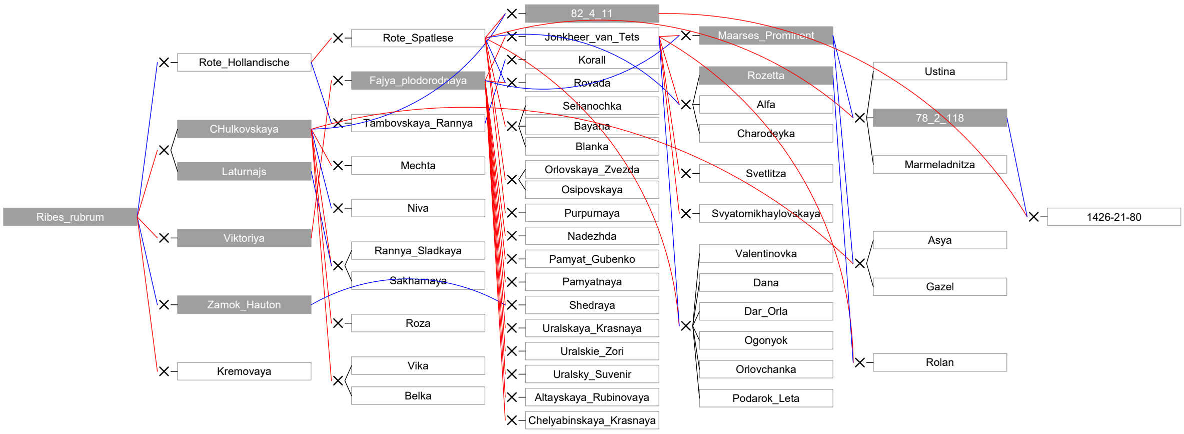

Figure S1. *Ribes rubrum* descendants. White rectangles show presented in this study genotypes. Grey rectangles show absented in this study genotypes. Figure has been created by Pedimap software.

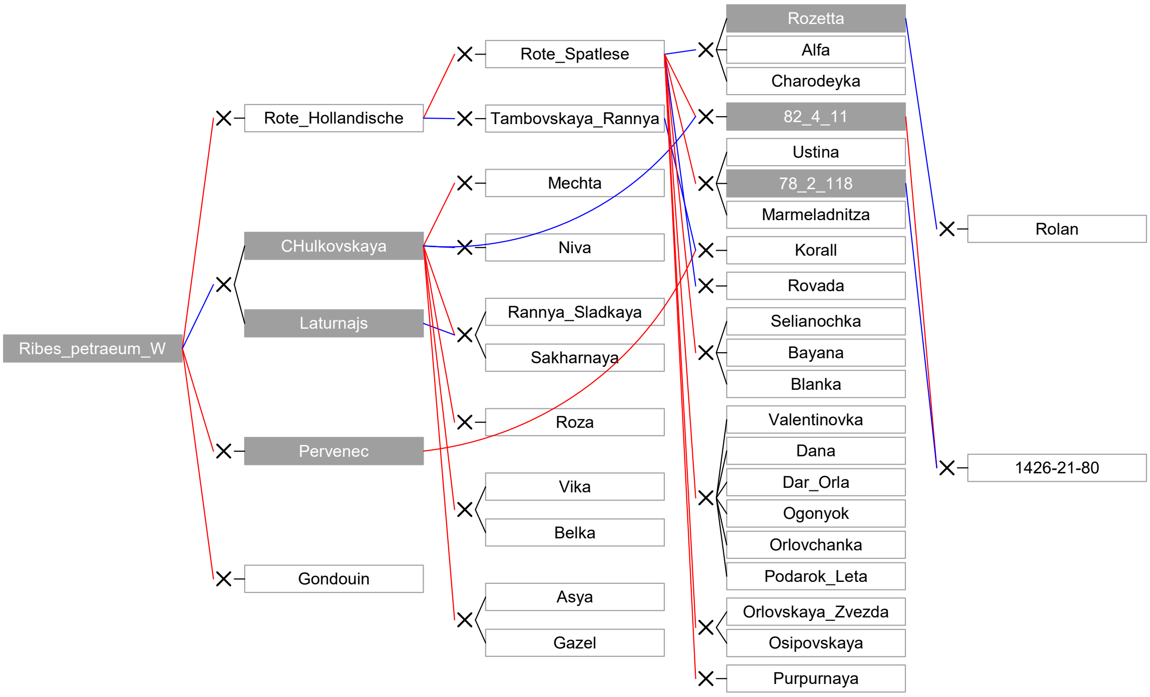

Figure S2. *Ribes petraeum* descendants (white rectangle shows genotype presented in this study, grey rectangle shows genotype missing in this study). Figure has been created by Pedimap software.

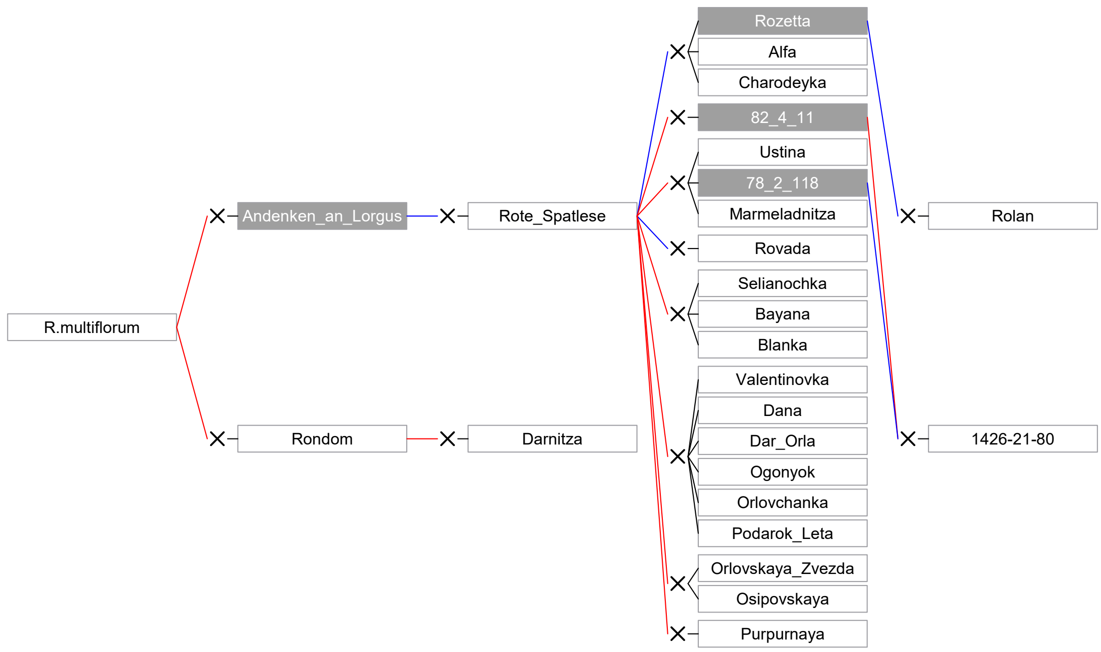

Figure S3. *Ribes multiflorum* descendants (white rectangle shows genotype presented in this study, grey rectangle shows genotype missing in this study). Figure has been created by Pedimap software.

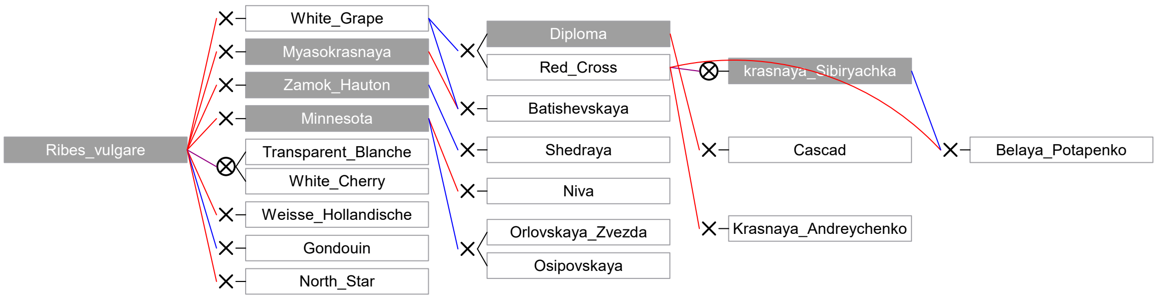

Figure S4. *Ribes vulgare* descendants (white rectangle shows genotype presented in this study, grey rectangle shows genotype missing in this study). Figure has been created by Pedimap software.

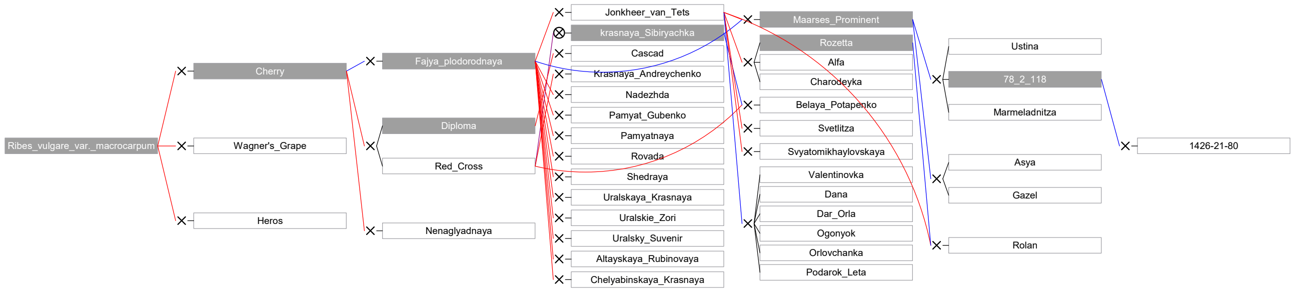

Figure S5. *Ribes vulgare* var *macrocarpum*descendants (white rectangle shows genotype presented in this study, grey rectangle shows genotype missing in this study). Figure has been created by Pedimap software.

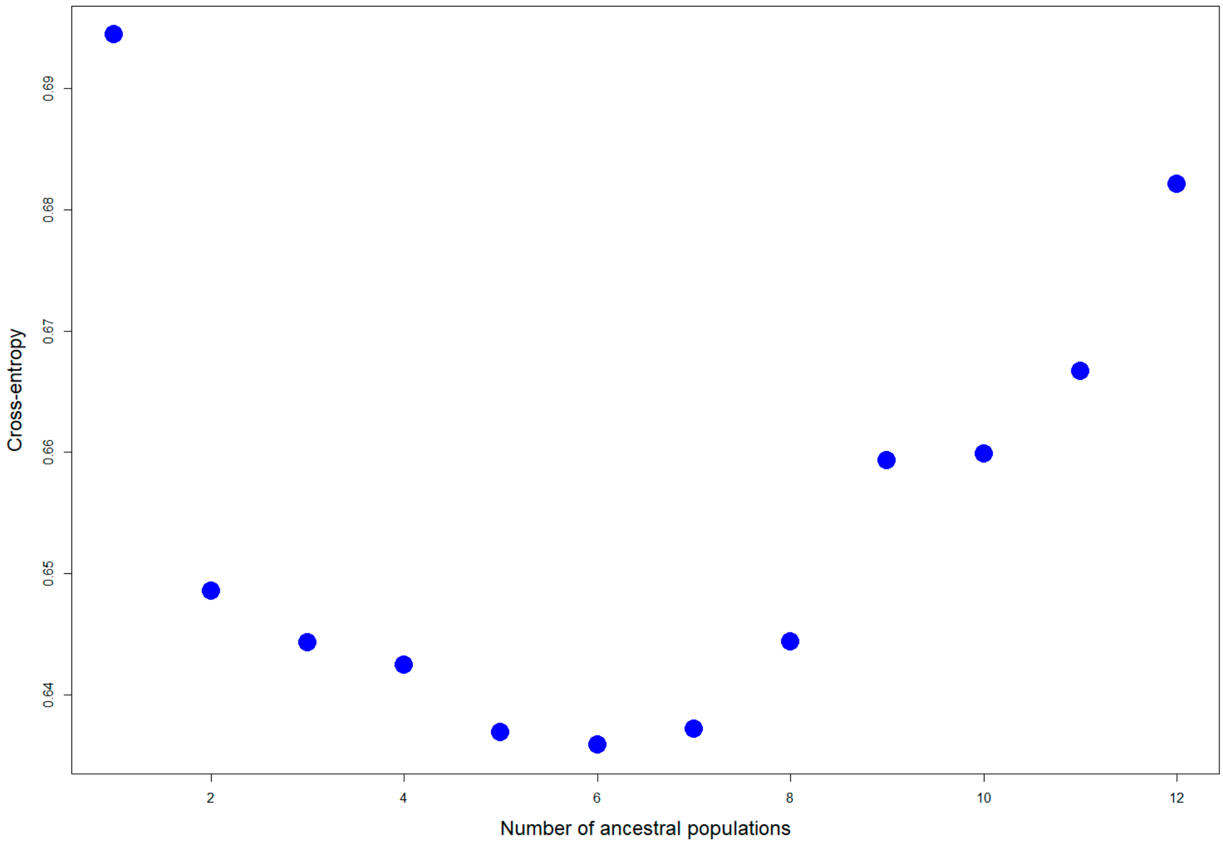

Figure S6. Determination of the appropriate number of clusters (K) based on the cross-entropy criterion by LEA software.

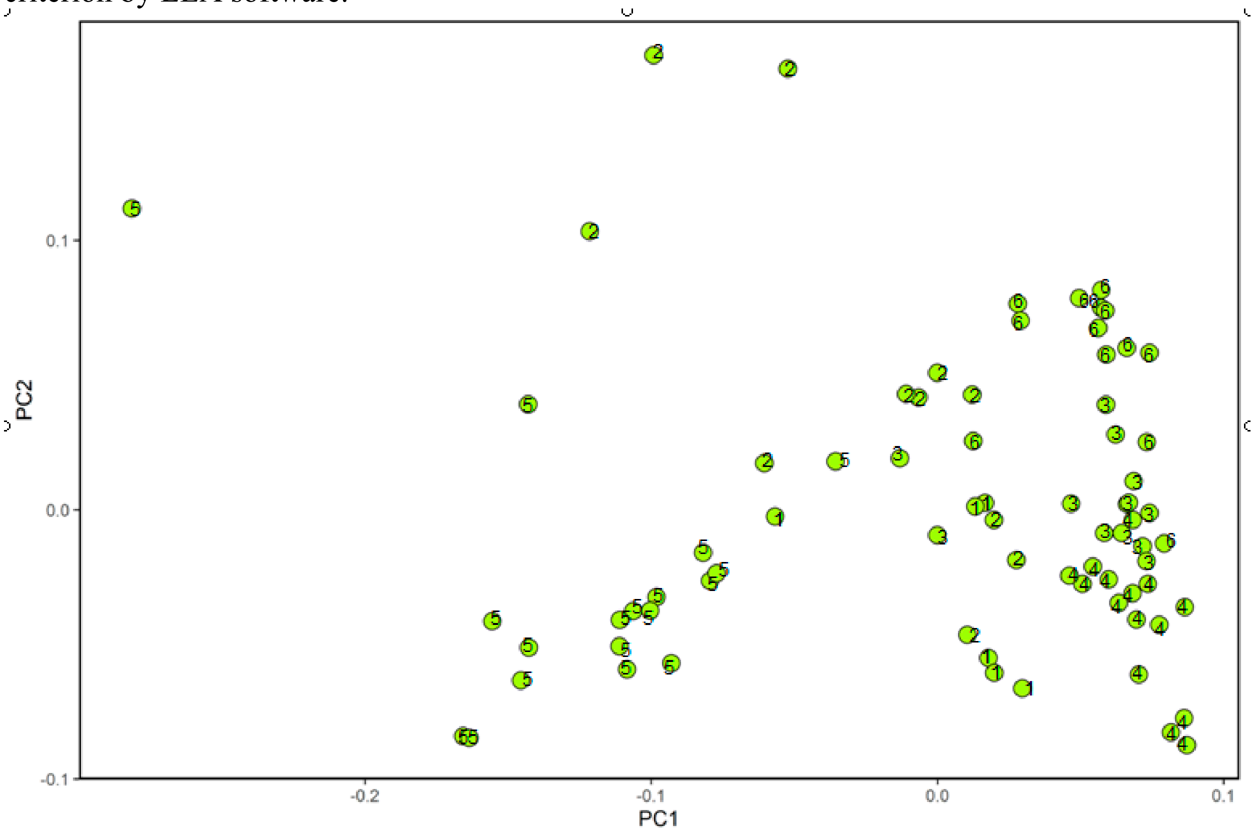

Figure S7. Admixture clusters designated on MDS plot. Numbers refer to population structure clusters.
